# Supplementary material for: Patient-specific models link neurotransmitter receptor mechanisms with motor and visuospatial axes of Parkinson’s disease
Source: Nat Commun. 2023 Sep 26;14:6009. doi: 10.1038/s41467-023-41677-w (PMC10522603; doi:10.1038/s41467-023-41677-w)
Supplement: Supplementary file 1 — Supplementary information [file 41467_2023_41677_MOESM1_ESM.pdf]

Patient-specific models link neurotransmitter receptor mechanisms with motor and visuospatial axes of Parkinson's disease

Ahmed Faraz Khan[1,2,3], Quadri Adewale[1,2,3], Sue-Jin Lin[1,2,3], Tobias R. Baumeister[1,2,3], Yashar Zeighami[1,4], Felix Carbonell[5], Nicola Palomero-Gallagher[6,7,8], Yasser Iturria-Medina[1,2,3]*

[1]Department of Neurology and Neurosurgery, Montreal Neurological Institute, McGill University, Montreal, Quebec, Canada

[2]McConnell Brain Imaging Center, Montreal Neurological Institute, Montreal, Canada

[3]Ludmer Centre for Neuroinformatics & Mental Health, Montreal, Canada

[4]Douglas Research Centre, Department of Psychiatry, McGill University, Montreal, Canada

[5]Biospective Inc., Montreal, Canada

[6]Institute of Neuroscience and Medicine (INM-1), Research Centre Jülich, Jülich, Germany

[7]Cécile and Oskar Vogt Institute of Brain Research, Medical Faculty, Heinrich-Heine University, Düsseldorf, Germany

[8]Department of Psychiatry, Psychotherapy, and Psychosomatics, Medical Faculty, RWTH Aachen, and JARA - Translational Brain Medicine, Aachen, Germany

* Correspondence to: Y I-M, 3801 University Street, room NW312, Montreal Neurological Institute, McGill University, Montreal, Canada H3A 2B4. Email: *yasser.iturriamedina@mcgill.ca*

Supplementary Table S1: *Summary of demographic data for N=71 PD patients.*

**Category**	**PD subjects**
**Mean MDS-UPDRS Part III score**	18.8 ± 8.7
**Female patients**	20 (28.2%)
**Mean age (years)**	59.6 ± 9.8
**Mean education (years)**	15.5 ± 2.8
**Non-white patients**	0
**Right handed patients**	64 (90.1%)

Supplementary Table S2: *Mean and standard deviation of the number of clinical evaluations per subject.*

**Category**	**Number of evaluations**
**BJLOT**	7.62 ± 1.13
**GDS**	8.27 ± 1.09
**HVLT**	7.65 ± 1.14
**LNS**	7.63 ± 1.12
**LXF**	0.79 ± 0.56
**NP1**	15.0 ± 1.9
**NP2**	15.0 ± 1.9
**NP3**	19.5 ± 4.0
**NP4**	9.94 ± 3.0
**MoCA**	7.63 ± 1.12
**SF**	7.62 ± 1.11
**STAIAD**	8.28 ± 1.08
**SDM**	7.66 ± 1.15

Supplementary Table S3: *Neurotransmitter receptor ligands used to obtain receptor maps.*

Neurotransmitter	Receptor	Ligand	Type
**Glutamate**	AMPA	[^{3}H]-AMPA	Agonist
	NMDA	[^{3}H]-MK-801	Antagonist
	Kainate	[^{3}H]-Kainate	Agonist
**GABA**	$GABA_A$	[^{3}H]-Muscimol	Agonist
	$GABA_B$	[^{3}H]-CGP 54626	Antagonist
	$GABA_A$-associated benzodiazepine binding site ($GABA_A$/BZ)	[^{3}H]-Flumazenil	Antagonist
**Acetylcholine**	M_1	[^{3}H]-Pirenzepine	Antagonist
	M_2	[^{3}H]-Oxotremorine-M	Agonist
	M_3	[^{3}H]-4-DAMP	Antagonist
	Nicotinic $\alpha_4\beta_2$	[^{3}H]-Epibatidine	Agonist
**Noradrenaline**	α_1	[^{3}H]-Prazosin	Antagonist
	α_2	[^{3}H]-RX 821002	Antagonist
**Serotonin**	5-HT_{1A}	[^{3}H]-8-OH-DPAT	Agonist
	5-HT_2	[^{3}H]-Ketanserin	Antagonist
**Dopamine**	D_1	[^{3}H]-SCH 23390	Antagonist

Supplementary Table S4: *Brain regions with receptor data, and the corresponding atlas used to extract the ROI map. Note that regions are defined by cytoarchitecture, and thus do not correspond perfectly with functional regions.*

Lobe	Anatomical subdivision	Jülich area	Region name	Atlas source
**Occipital lobe**	Visual cortex	hOc1	Brodmann's area 17 / V1	Jülich
		hOc2	Brodmann's area 18 / V2	Jülich
		hOc4d	V4	Jülich
		hOc3a	V3a	Jülich
		hOc3d	V3d	Jülich
		hOc3v	V3v	Jülich
		hOc4v	V4	Jülich
	Extrastriate cortex	FG1	Part of Brodmann area 19	Jülich
		FG2	Part of Brodmann area 19	Jülich
**Parietal lobe**	Somatosensory cortex	1	Brodmann's area 1	Jülich
		2	Brodmann's area 2	Jülich
		3a	Brodmann's area 3a	Jülich
		3b	Brodmann's area 3b	Jülich
	Superior parietal lobule	5L	Brodmann's area 5L	Jülich
		5M	Brodmann's area 5M	Jülich
		7A	Brodmann's area 7A	Jülich
	Inferior parietal lobule	PGa	Anterior inferior parietal area	Jülich
		PGp	Posterior inferior parietal area	Jülich
		PFt	Temporal inferior parietal	Jülich

			area	
		PFm	Medial inferior parietal area	Jülich
**Temporal lobe**	Auditory cortex	Te1	Temporal area 1 (part of Brodmann's area 41)	Jülich
		Te2	Temporal area 2 (part of Brodmann's area 41)	Jülich
	Hippocampus	CA	Cornu ammonis	Jülich
		DG	Dentate gyrus	Jülich
	Subiculum	Subiculum	Subiculum	Jülich
	Entorhinal cortex	Ent	Brodmann's area 28	Jülich
		20	Brodmann's area 20	Brodmann
		21	Brodmann's area 21	Brodmann
		22	Brodmann's area 22	Brodmann
		36	Brodmann's area 36	Brodmann
		37	Brodmann's area 37	Brodmann
		38	Brodmann's area 38	Brodmann
**Frontal lobe**	Agranular premotor cortex	6	Brodmann's area 6	Jülich
	Primary motor cortex	4p	Brodmann's area 4p	Jülich
	Broca's region	44		Jülich
		45		Jülich
	Frontopolar cortex	Fp1	Frontopolar area (part of Brodmann area 10)	Jülich
		Fp2	Frontopolar area (part of Brodmann area	Jülich

			10)	
	Orbitofrontal cortex	Fo1	Orbitofrontal area (part of Brodmann area 11)	Jülich
	Lateral prefrontal	46	Brodmann's area 46	Brodmann
		47	Brodmann's area 47	Brodmann
		8	Brodmann's area 8	Brodmann
		9	Brodmann's area 9	Brodmann
**Cingulate regions (multiple lobes)**	Anterior cingulate	p24ab	Pregenual cingulate areas p24a & p24b	Jülich
		p32	Pregenual cingulate area p32	Jülich
	Posterior cingulate	23	Brodmann's area 23	Brodmann
		31	Brodmann's area 31	Brodmann
**Basal ganglia**	Striatum	Putamen	Putamen	AAL
		Caudate	Caudate nucleus	AAL
	Pallidum	Globus pallidus	Globus pallidus	DISTAL
	Subthalamic nucleus	STN	Subthalamic nucleus	DISTAL
**Forebrain**	Thalamus	Thalamus (anterior)	Thalamus (anterior)	AAL
		Thalamus (medial)	Thalamus (medial)	AAL
		Thalamus (lateral)	Thalamus (lateral)	AAL

Supplementary Table S5: *Biological parameters most correlated with clinical symptoms in PD via the primary component, and the percentage of clinical score covariance explained via this component.*

Neuroimaging Modality	Model Parameter	Receptor Type	Explained Variance
GM	AMPA x fALLF	Glutamatergic	0.12%
	$GABA_B$	GABAergic	0.18%
	$\alpha_4\beta_2$ x GM	Cholinergic	0.31%
	M_1 x fALLF	Cholinergic	0.10%
	M_2 x fALLF	Cholinergic	0.31%
	$\alpha_4\beta_2$ x fALLF	Cholinergic	0.10%
	M_3 x FA	Cholinergic	0.16%
	M_1 x t1/t2	Cholinergic	0.12%
	M_1	Cholinergic	0.10%
	α_2 x fALLF	Adrenergic	0.42%
	$5HT_{1A}$ x SPECT	Serotonergic	0.29%
	D_1 x fALLF	Dopaminergic	0.29%
	GM	Non-Receptor	0.14%
	SPECT	Non-Receptor	0.20%
fALFF	Kainate x SPECT	Glutamatergic	0.10%
	NMDA x FA	Glutamatergic	0.33%
	Kainate x t1/t2	Glutamatergic	0.24%
	Bz site x GM	GABAergic	0.31%
	$GABA_B$ x fALLF	GABAergic	0.29%
	$GABA_A$ x FA	GABAergic	0.27%
	M_2 x FA	Cholinergic	0.13%
	M_1	Cholinergic	0.18%
	M_2	Cholinergic	0.21%
	$5HT_{1A}$ x t1/t2	Serotonergic	0.08%
	D_1 x GM	Dopaminergic	0.19%
	GM	Non-Receptor	0.16%
SPECT	Kainate x FA	Glutamatergic	0.15%
	α_1 x FA	Adrenergic	0.12%
	$5HT_2$ x GM	Serotonergic	0.18%
	$5HT_{1A}$ x MD	Serotonergic	0.25%
	$5HT_2$ x t1/t2	Serotonergic	0.23%
	$5HT_2$	Serotonergic	0.21%
	D_1 x FA	Dopaminergic	0.14%
	D_1	Dopaminergic	0.21%
	FA	Non-Receptor	0.31%
	MD	Non-Receptor	0.19%

FA	Kainate x GM	Glutamatergic	0.22%
	AMPA x t1/t2	Glutamatergic	0.13%
	Kainate x t1/t2	Glutamatergic	0.18%
	AMPA	Glutamatergic	0.36%
	Kainate	Glutamatergic	0.28%
	$GABA_A$ x MD	GABAergic	0.20%
	Bz site x t1/t2	GABAergic	0.20%
	$GABA_B$ x t1/t2	GABAergic	0.30%
	$GABA_A$	GABAergic	0.67%
	M_1 x fALLF	Cholinergic	0.15%
	M_3 x MD	Cholinergic	0.48%
	$\alpha_4\beta_2$ x t/1t2	Cholinergic	0.19%
	α_2 x GM	Adrenergic	0.25%
	α_2 x MD	Adrenergic	0.23%
	α_1 x t1/t2	Adrenergic	0.16%
	α_1	Adrenergic	0.13%
	$5HT_{1A}$ x SPECT	Serotonergic	0.08%
	$5HT_2$ x SPECT	Serotonergic	0.18%
	$5HT_2$ x MD	Serotonergic	0.17%
	$5HT_2$ x t1/t2	Serotonergic	0.12%
	GM	Non-Receptor	0.28%
	SPECT	Non-Receptor	0.12%
	MD	Non-Receptor	0.21%
	t1/t2	Non-Receptor	0.23%
	spreading	Non-Receptor	0.17%
MD	AMPA x fALLF	Glutamatergic	0.23%
	Kainate x fALLF	Glutamatergic	0.37%
	Kainate x FA	Glutamatergic	0.20%
	NMDA x MD	Glutamatergic	0.25%
	Kainate x MD	Glutamatergic	0.38%
	$GABA_A$ x GM	GABAergic	0.41%
	Bz site x fALLF	GABAergic	0.31%
	$GABA_B$ x MD	GABAergic	0.11%
	$GABA_A$	GABAergic	0.35%
	Bz site	GABAergic	0.50%
	M_1 x MD	Cholinergic	0.18%
	M_1	Cholinergic	0.21%
	M_2	Cholinergic	0.43%
	M_3	Cholinergic	0.67%
	α_1 x FA	Adrenergic	0.10%
	α_2	Adrenergic	0.13%

	$5HT_{1A}$ x GM	Serotonergic	0.12%
	$5HT_2$ x fALLF	Serotonergic	0.54%
	$5HT_{1A}$ x FA	Serotonergic	0.22%
	$5HT_2$	Serotonergic	0.45%
	D_1 x FA	Dopaminergic	0.15%
	fALLF	Non-Receptor	0.29%
	FA	Non-Receptor	0.24%
t1/t2	AMPA x FA	Glutamatergic	0.21%
	NMDA x FA	Glutamatergic	0.55%
	NMDA	Glutamatergic	0.38%
	Kainate	Glutamatergic	0.16%
	Bz site x GM	GABAergic	0.31%
	$GABA_A$ x FA	GABAergic	0.62%
	Bz site x FA	GABAergic	0.27%
	Bz site x MD	GABAergic	0.35%
	Bz site x t1/t2	GABAergic	0.13%
	$GABA_B$ x t1/t2	GABAergic	0.20%
	M_1 x SPECT	Cholinergic	0.21%
	M_1 x FA	Cholinergic	0.15%
	M_2 x FA	Cholinergic	0.08%
	M_1 x t1/t2	Cholinergic	0.13%
	M_3 x t1/t2	Cholinergic	0.18%
	α_2 x fALLF	Adrenergic	0.28%
	α_2	Adrenergic	0.21%
	$5HT_2$ x GM	Serotonergic	0.52%
	$5HT_2$ x SPECT	Serotonergic	0.14%
	D_1 x FA	Dopaminergic	0.19%
	offset	Non-Receptor	0.25%
	FA	Non-Receptor	0.19%

Supplementary Table S6: *Biological parameters most correlated with clinical symptoms in PD via the secondary component, and the percentage of clinical score covariance explained.*

Neuroimaging Modality	Model Parameter	Receptor Type	Explained Variance
GM	NMDA x GM	Glutamatergic	0.06%
	NMDA x SPECT	Glutamatergic	0.10%
	NMDA x MD	Glutamatergic	0.05%
	Kainate x MD	Glutamatergic	0.04%
	AMPA	Glutamatergic	0.08%
	NMDA	Glutamatergic	0.04%

	$GABA_A$ x GM	GABAergic	0.04%
	$GABA_A$ x fALLF	GABAergic	0.09%
	$GABA_A$ x FA	GABAergic	0.05%
	$GABA_A$ x MD	GABAergic	0.05%
	M_1 x fALLF	Cholinergic	0.07%
	M_1 x MD	Cholinergic	0.19%
	M_3	Cholinergic	0.08%
	$\alpha_4\beta_2$	Cholinergic	0.08%
	α_2 x SPECT	Adrenergic	0.11%
	α_1	Adrenergic	0.07%
	$5HT_2$ x FA	Serotonergic	0.13%
	$5HT_2$	Serotonergic	0.06%
	D_1 x GM	Dopaminergic	0.10%
	D_1 x MD	Dopaminergic	0.07%
	D_1	Dopaminergic	0.06%
	offset	Non-Receptor	0.06%
	t1/t2	Non-Receptor	0.04%
fALFF	$GABA_B$ x SPECT	GABAergic	0.06%
	M_3 x GM	Cholinergic	0.06%
	$\alpha_4\beta_2$ x t1/t2	Cholinergic	0.05%
	$5HT_2$ x MD	Serotonergic	0.08%
	$5HT_{1A}$ x t1/t2	Serotonergic	0.03%
	GM	Non-Receptor	0.04%
	FA	Non-Receptor	0.06%
	t1/t2	Non-Receptor	0.04%
SPECT	AMPA	Glutamatergic	0.04%
	NMDA	Glutamatergic	0.08%
	Kainate	Glutamatergic	0.12%
	$GABA_B$ x GM	GABAergic	0.05%
	Bz site x fALLF	GABAergic	0.05%
	$GABA_B$ x FA	GABAergic	0.07%
	$\alpha_4\beta_2$ x SPECT	Cholinergic	0.14%
	α_1 x SPECT	Adrenergic	0.04%
	α_2 x SPECT	Adrenergic	0.07%
	α_2	Adrenergic	0.09%
	$5HT_2$ x fALLF	Serotonergic	0.09%
	D_1 x FA	Dopaminergic	0.03%
FA	Kainate x GM	Glutamatergic	0.10%
	NMDA x SPECT	Glutamatergic	0.10%
	Kainate x FA	Glutamatergic	0.09%
	Kainate	Glutamatergic	0.12%

	$GABA_A$ x SPECT	GABAergic	0.04%
	$GABA_B$	GABAergic	0.07%
	α_1 x GM	Adrenergic	0.06%
	α_2 x fALLF	Adrenergic	0.05%
	α_1 x SPECT	Adrenergic	0.09%
	α_1 x t1/t2	Adrenergic	0.06%
	$5HT_{1A}$	Serotonergic	0.08%
	D_1 x MD	Dopaminergic	0.07%
MD	Kainate x MD	Glutamatergic	0.04%
	Bz site x fALLF	GABAergic	0.06%
	Bz site x SPECT	GABAergic	0.07%
	$GABA_A$ x FA	GABAergic	0.07%
	Bz site x FA	GABAergic	0.04%
	$GABA_B$ x MD	GABAergic	0.16%
	Bz site	GABAergic	0.06%
	$GABA_B$	GABAergic	0.06%
	M_2 x fALLF	Cholinergic	0.05%
	M_1 x SPECT	Cholinergic	0.03%
	M_2 x FA	Cholinergic	0.07%
	M_1 x t1/t2	Cholinergic	0.06%
	M_2 x t1/t2	Cholinergic	0.06%
	M_2	Cholinergic	0.06%
	M_3	Cholinergic	0.09%
	α_2 x fALLF	Adrenergic	0.04%
	$5HT_{1A}$ x GM	Serotonergic	0.05%
	$5HT_{1A}$ x t1/t2	Serotonergic	0.04%
	$5HT_2$	Serotonergic	0.06%
	D_1 x SPECT	Dopaminergic	0.07%
	t1/t2	Non-Receptor	0.06%
t1/t2	AMPA x SPECT	Glutamatergic	0.07%
	NMDA x FA	Glutamatergic	0.07%
	$GABA_A$ x GM	GABAergic	0.04%
	$GABA_B$ x GM	GABAergic	0.04%
	Bz site x SPECT	GABAergic	0.06%
	$GABA_B$ x SPECT	GABAergic	0.05%
	M_1 x GM	Cholinergic	0.08%
	M_3 x GM	Cholinergic	0.14%
	M_2 x SPECT	Cholinergic	0.04%
	$\alpha_4\beta_2$ x MD	Cholinergic	0.06%
	M_1	Cholinergic	0.16%
	M_3	Cholinergic	0.15%

	α_1 x GM	Adrenergic	0.03%
	α_1 x SPECT	Adrenergic	0.07%
	α_1 x FA	Adrenergic	0.04%
	α_1 x MD	Adrenergic	0.08%
	$5HT_2$ x GM	Serotonergic	0.07%
	$5HT_2$ x MD	Serotonergic	0.05%
	D_1 x MD	Dopaminergic	0.06%
	D_1 x t1/t2	Dopaminergic	0.07%
	GM	Non-Receptor	0.06%

Supplementary Table S7: *Total MCM parameter-clinical co-variance explained by receptor type in PD patients (via each SVD component).*

Receptor Type	Total variance explained in the primary component	Total variance explained in the secondary component
**Glutamatergic**	4.85%	1.19%
**GABAergic**	5.97%	1.24%
**Cholinergic**	4.77%	1.74%
**Adrenergic**	2.02%	0.88%
**Serotonergic**	3.77%	0.75%
**Dopaminergic**	1.16%	0.53%

Supplementary Table S8: *Performance gain due to the inclusion of receptor maps, and the p-value from a two-sample t-test for each modality, across all (N=71) subjects.*

Imaging Modality	Average Gain in R^2	P-value
**GM**	35.6% ± 10.8%	$P=1.16\times10^{-27}$
**fALFF**	18.8% ± 8.0%	$P=7.22\times10^{-13}$
**SPECT**	20.2% ± 12.4%	$P=1.38\times10^{-9}$
**FA**	21.7% ± 11.8%	$P=5.87\times10^{-11}$
**MD**	19.0% ± 9.1%	$P=1.69\times10^{-9}$
**t1/t2**	17.1% ± 9.3%	$P=5.83\times10^{-9}$

Supplementary Table S9: *Performance gain due to true receptor distributions over null maps, and p-value of the true receptor data model belonging to the null distribution, using Fisher's method to combine p-values across all (N=71) subjects.*

Imaging Modality	Average Gain in R^2	P-value
**GM**	13.4% ± 5.3%	$P=2\times10^{-16}$
**fALFF**	7.3% ± 4.5%	$P=2\times10^{-16}$
**SPECT**	6.7% ± 4.3%	$P=2\times10^{-16}$
**FA**	7.5% ± 5.3%	$P=2\times10^{-16}$
**MD**	5.3% ± 4.0%	$P=2\times10^{-16}$
**t1/t2**	6.0% ± 3.5%	$P=2\times10^{-16}$

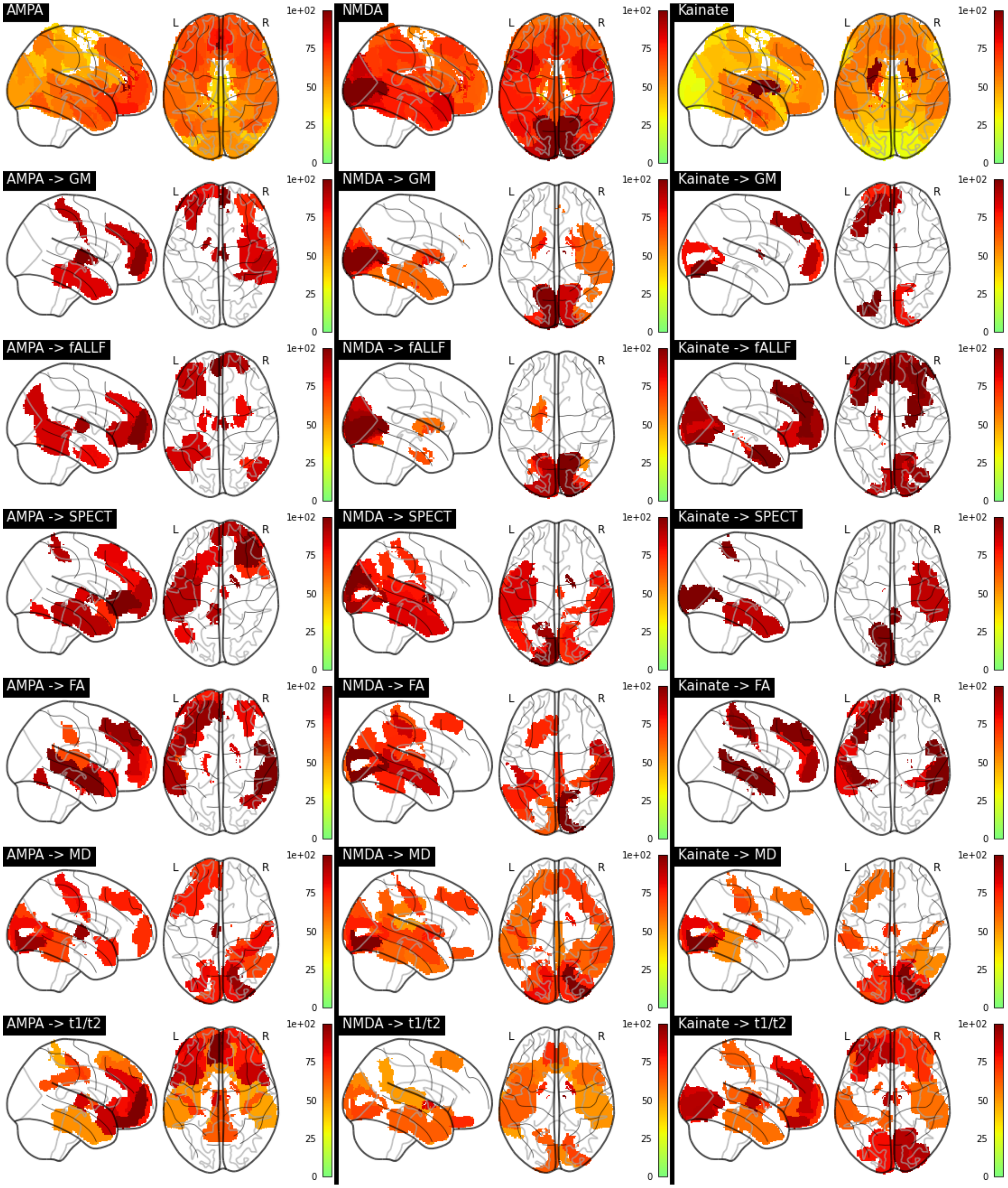

Supplementary Figure S1: *Glutamatergic receptor influence maps. The first row shows the densities of AMPA, NMDA and kainate receptors, with remaining rows showing their influence on gray matter density (GM), fractional amplitude of low frequency fluctuations (fALFF) from rs-fMRI, dopaminergic transporter (DAT) SPECT density, fractional anisotropy (FA), mean diffusivity (MD) and t1/t2 ratio. All maps are re-scaled to arbitrary units for visualization, and show only regions with significant z-scores (P<0.05) of Wilcoxon rank-sum statistics relative to the Wilcoxon statistics due to null distributions (1000 iterations with permuted receptor maps).*

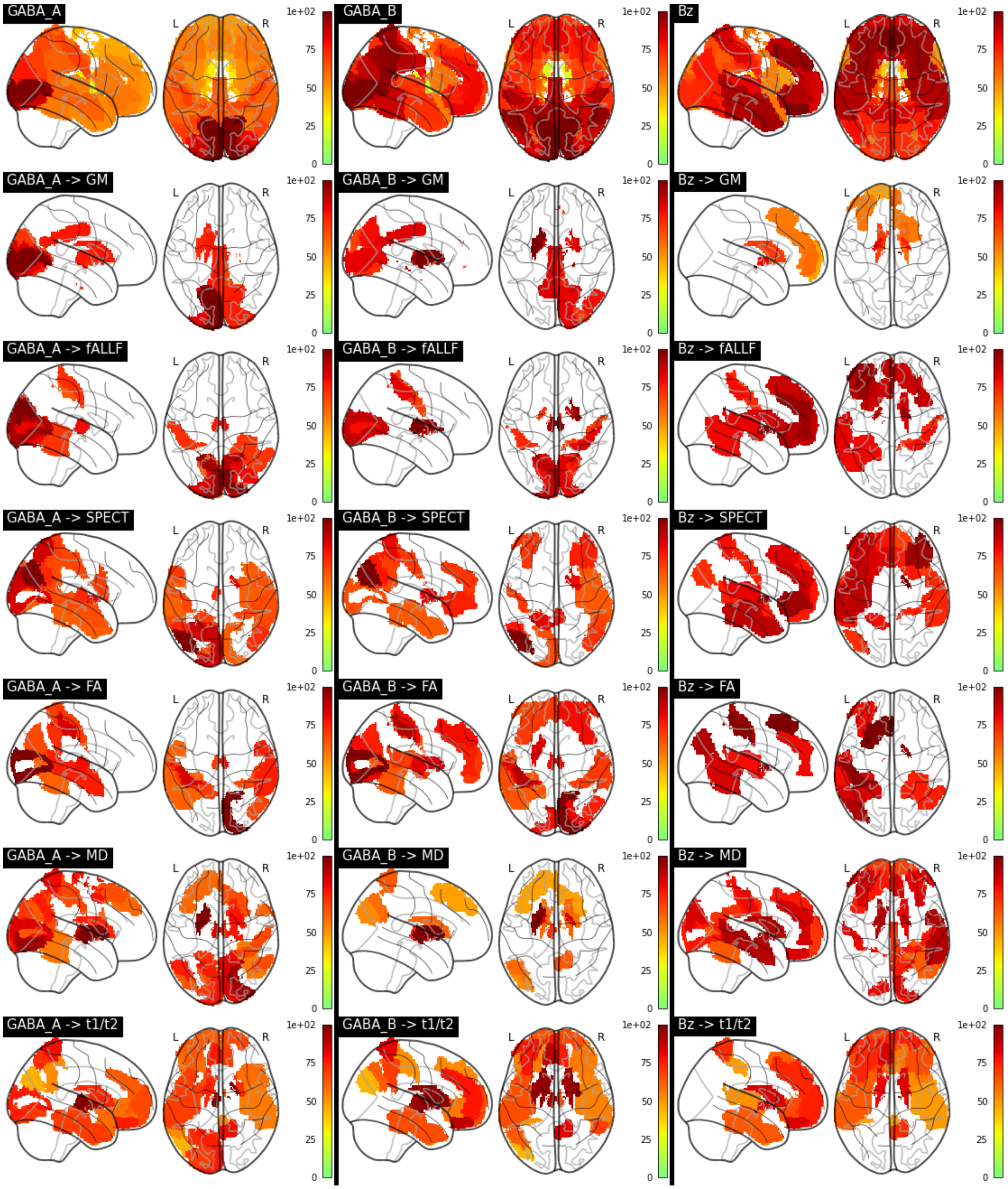

Supplementary Figure S2: *GABAergic receptor influence on imaging modalities. The first row shows density maps for* $GABA_A$*,* $GABA_B$ *and the Bz binding site, with remaining rows showing receptor influence maps for each imaging modality, including gray matter density (GM), fractional amplitude of low frequency fluctuations (fALFF) from rs-fMRI, dopaminergic transporter (DAT) SPECT density, fractional anisotropy (FA), mean diffusivity (MD) and t1/t2 ratio. All maps are re-scaled to arbitrary units for visualization, and show only regions with significant z-scores (P<0.05) of Wilcoxon rank-sum statistics relative to the Wilcoxon statistics due to null distributions (1000 iterations with permuted receptor maps).*

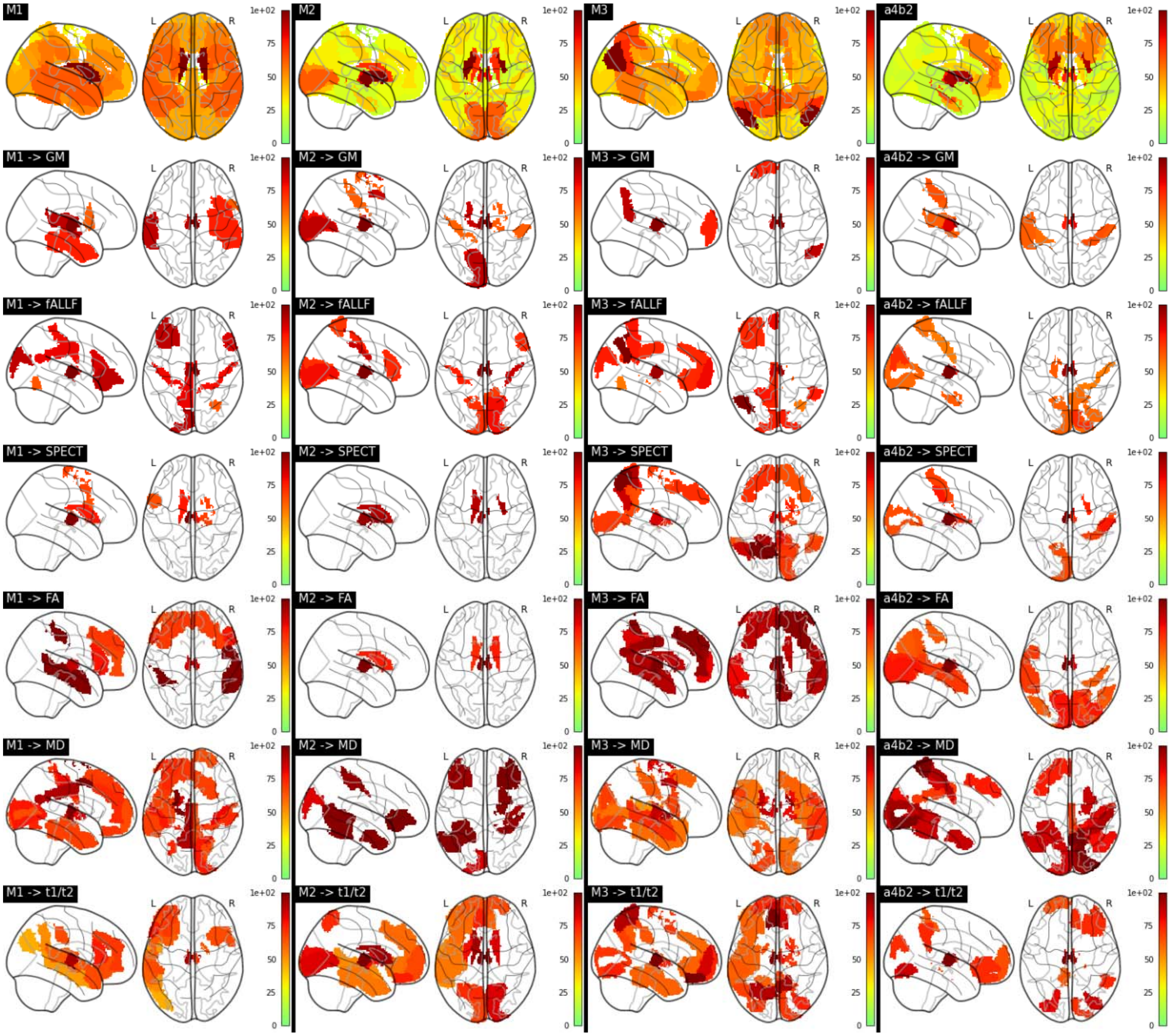

Supplementary Figure S3: *Cholinergic receptor influence maps. The first row shows the densities of the muscarinic M_1, M_2 and M3, and cholinergic $\alpha_4\beta_2$ receptors, with remaining rows showing receptor influence maps for each imaging modality, including gray matter density (GM), fractional amplitude of low frequency fluctuations (fALFF) from rs-fMRI, dopaminergic transporter (DAT) SPECT density, fractional anisotropy (FA), mean diffusivity (MD) and t1/t2 ratio. All maps are re-scaled to arbitrary units for visualization, and show only regions with significant z-scores ($P<0.05$) of Wilcoxon rank-sum statistics relative to the Wilcoxon statistics due to null distributions (1000 iterations with permuted receptor maps).*

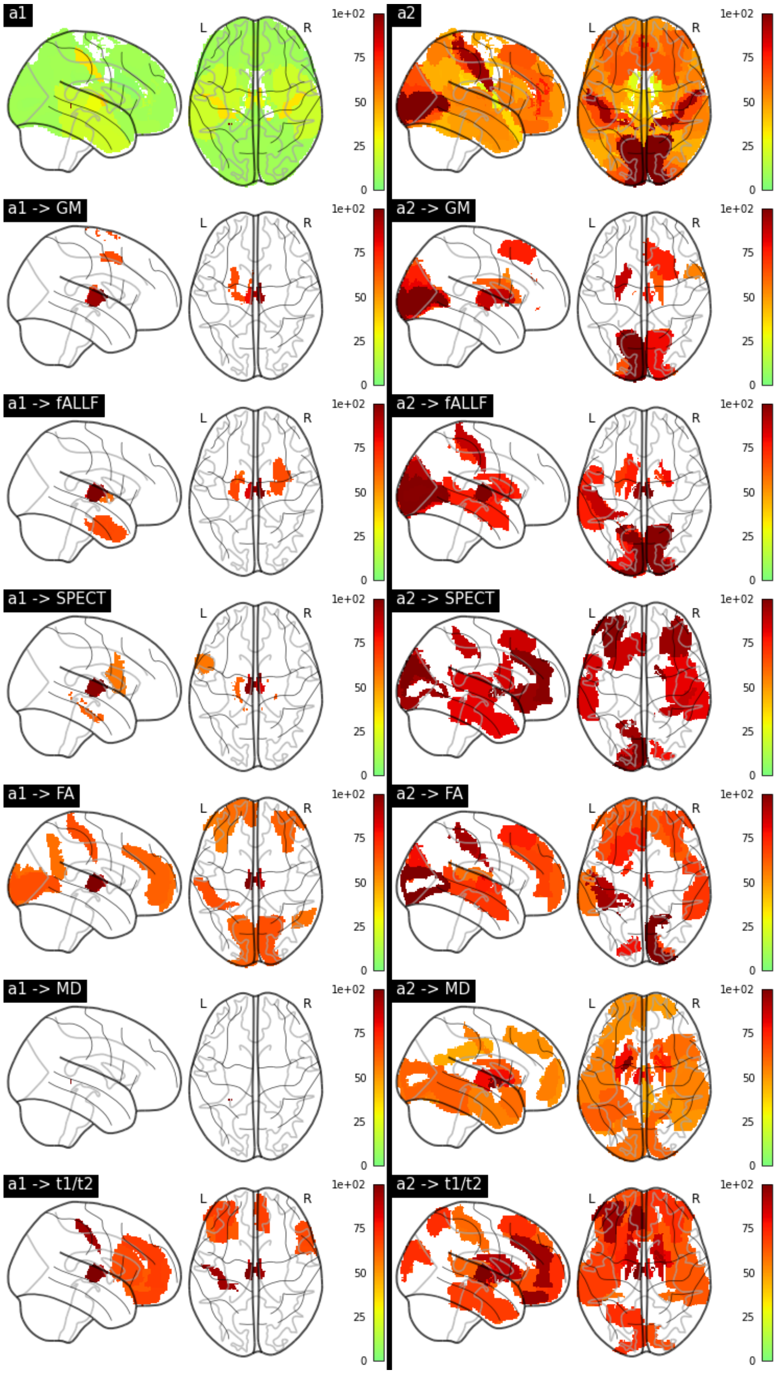

Supplementary Figure S4: *Adrenergic receptor influence maps. The first row shows* α_1 *and* α_2 *receptor density maps. with remaining rows showing receptor influence maps for each imaging modality, including gray matter density (GM), fractional amplitude of low frequency fluctuations (fALFF) from rs-fMRI, dopaminergic transporter (DAT) SPECT density, fractional anisotropy (FA), mean diffusivity (MD) and t1/t2 ratio. All maps are re-scaled to arbitrary units for visualization, and show only regions with significant z-scores (P<0.05) of Wilcoxon rank-sum statistics relative to the Wilcoxon statistics due to null distributions (1000 iterations with permuted receptor maps).*

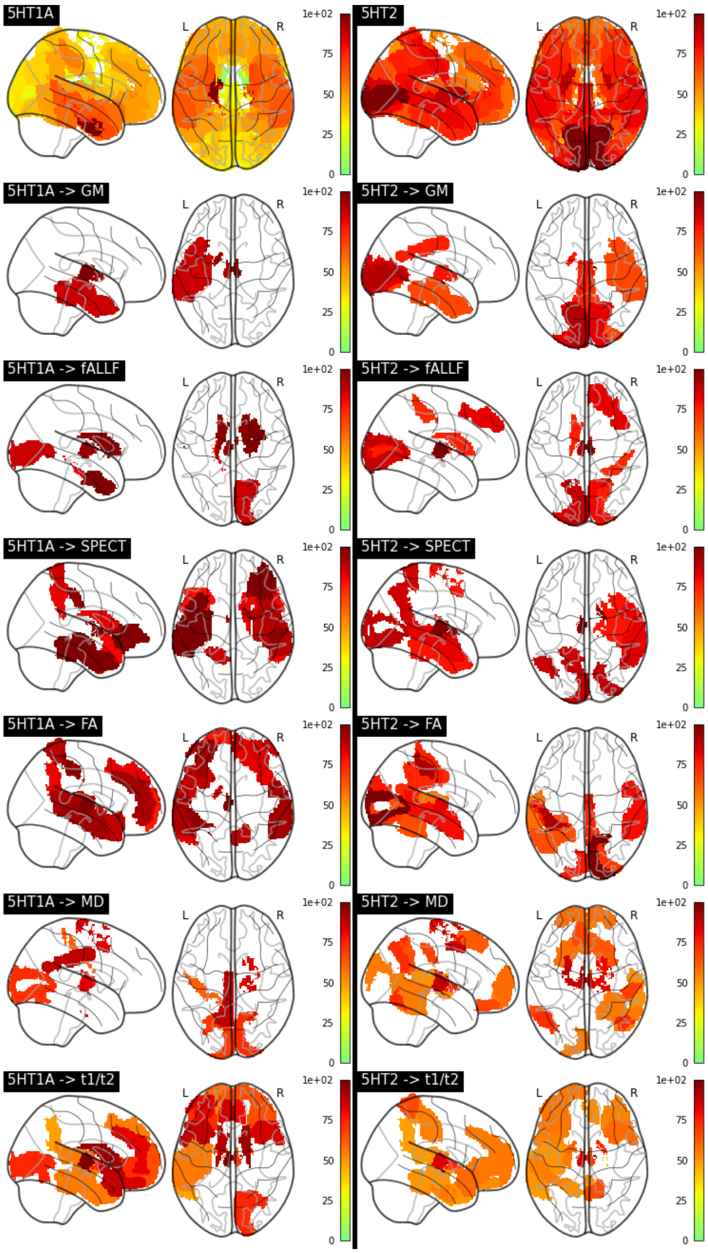

Supplementary Figure S5: *Serotonergic receptor influence maps. The first row shows the $5HT_{1A}$ and $5HT_2$ serotonergic receptor density maps, with remaining rows showing receptor influence maps on gray matter density (GM), fractional amplitude of low frequency fluctuations (fALFF) from rs-fMRI, dopaminergic transporter (DAT) SPECT density, fractional anisotropy (FA), mean diffusivity (MD) and t1/t2 ratio. All maps are re-scaled to arbitrary units for visualization, and show only regions with significant z-scores ($P<0.05$) of Wilcoxon rank-sum statistics relative to the Wilcoxon statistics due to null distributions (1000 iterations with permuted receptor maps).*

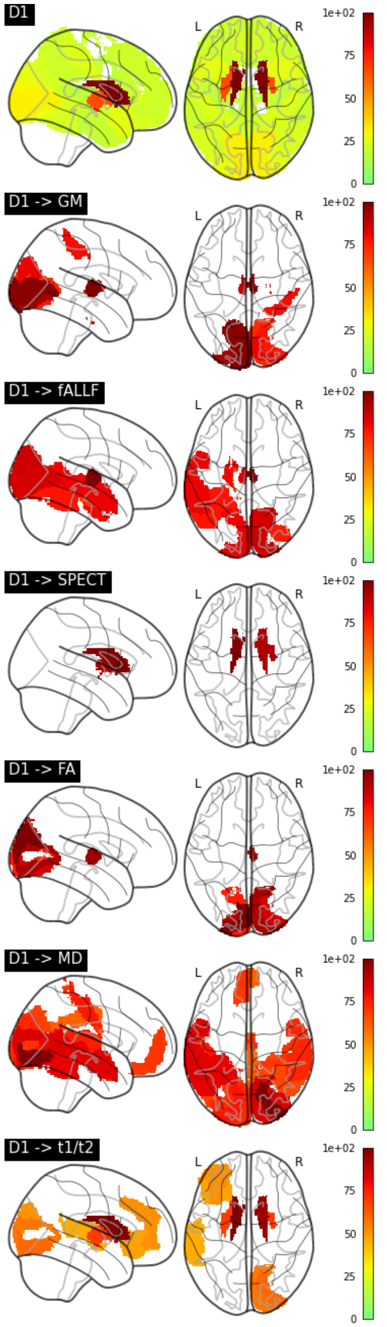

Supplementary Figure S6: *Dopaminergic receptor influence maps. The first row shows the D_1 dopaminergic receptor density, with remaining rows showing its influence on each imaging modality, which include gray matter density (GM), fractional amplitude of low frequency fluctuations (fALFF) from rs-fMRI, dopaminergic transporter (DAT) SPECT density, fractional anisotropy (FA), mean diffusivity (MD) and t1/t2 ratio. All maps are re-scaled to arbitrary units for visualization, and show only regions with significant z-scores ($P<0.05$) of Wilcoxon rank-sum statistics relative to the Wilcoxon statistics due to null distributions (1000 iterations with permuted receptor maps).*
