# Supplementary material for: Patient-specific models link neurotransmitter receptor mechanisms with motor and visuospatial axes of Parkinson’s disease
Source: Nat Commun. 2023 Sep 26;14:6009. doi: 10.1038/s41467-023-41677-w (PMC10522603; doi:10.1038/s41467-023-41677-w)
Supplement: Supplementary file 3 — Reporting Summary [file 41467_2023_41677_MOESM3_ESM.pdf]

nature portfolio

Corresponding author(s): Yasser Iturria Medina

Last updated by author(s): August 17, 2023

Reporting Summary

Nature Portfolio wishes to improve the reproducibility of the work that we publish. This form provides structure for consistency and transparency in reporting. For further information on Nature Portfolio policies, see our Editorial Policies and the Editorial Policy Checklist.

Statistics

For all statistical analyses, confirm that the following items are present in the figure legend, table legend, main text, or Methods section.

n/a	Confirmed	
☐	☒	The exact sample size (*n*) for each experimental group/condition, given as a discrete number and unit of measurement
☐	☒	A statement on whether measurements were taken from distinct samples or whether the same sample was measured repeatedly
☐	☒	The statistical test(s) used AND whether they are one- or two-sided<br>*Only common tests should be described solely by name; describe more complex techniques in the Methods section.*
☐	☒	A description of all covariates tested
☐	☒	A description of any assumptions or corrections, such as tests of normality and adjustment for multiple comparisons
☐	☒	A full description of the statistical parameters including central tendency (e.g. means) or other basic estimates (e.g. regression coefficient) AND variation (e.g. standard deviation) or associated estimates of uncertainty (e.g. confidence intervals)
☐	☒	For null hypothesis testing, the test statistic (e.g. *F*, *t*, *r*) with confidence intervals, effect sizes, degrees of freedom and *P* value noted<br>*Give P values as exact values whenever suitable.*
☒	☐	For Bayesian analysis, information on the choice of priors and Markov chain Monte Carlo settings
☒	☐	For hierarchical and complex designs, identification of the appropriate level for tests and full reporting of outcomes
☐	☒	Estimates of effect sizes (e.g. Cohen's *d*, Pearson's *r*), indicating how they were calculated

Our web collection on statistics for biologists contains articles on many of the points above.

Software and code

Policy information about availability of computer code

Data collection	No software was used to collect data; it was downloaded from online databases. Neuroimaging data was pre-processed for analysis using FSL (release 6.0, https://fsl.fmrib.ox.ac.uk/fsl/fslwiki), SPM12 (version 12, http://fil.ion.ucl.ac.uk/spm), ComBat (commit 91f8bf3, https://github.com/Jfortin1/ComBatHarmonization) and DSI-Studio (March 8, 2019 build, https://dsi-studio.labsolver.org/).
Data analysis	Custom code in MATLAB 2019b (Update 3) was used to fit the models and perform the analysis will be incorporated into our lab's existing open-access toolbox (https://www.neuropm-lab.com/neuropm-box.html). The code for SVD-PLS analysis is available at https://www.neuropm-lab.com/publication-codes.html.

For manuscripts utilizing custom algorithms or software that are central to the research but not yet described in published literature, software must be made available to editors and reviewers. We strongly encourage code deposition in a community repository (e.g. GitHub). See the Nature Portfolio guidelines for submitting code & software for further information.

Data

Policy information about availability of data

All manuscripts must include a data availability statement. This statement should provide the following information, where applicable:

- Accession codes, unique identifiers, or web links for publicly available datasets
- A description of any restrictions on data availability
- For clinical datasets or third party data, please ensure that the statement adheres to our policy

The three datasets used in this study are publicly available. The PPMI database (neuroimaging and clinical evaluations; https://www.ppmi-info.org/) is available to access after completing a data use agreement and submitting an online application (https://www.ppmi-info.org/access-data-specimens/download-data). The HCP dataset (HCP-1065 [Yeh et al., NeuroImage, 2018]; tractography template for connectivity estimation; http://www.humanconnectomeproject.org/) is available at https://brain.labsolver.org/hcp_template.html, and receptor autoradiography data published in [Zilles & Palomero-Gallagher, Frontiers in Neuroanatomy, 2017] is available at https://github.com/AlGoulas/receptor_principles.

Research involving human participants, their data, or biological material

Policy information about studies with human participants or human data. See also policy information about sex, gender (identity/presentation), and sexual orientation and race, ethnicity and racism.

Reporting on sex and gender	Self-reported sex was considered as a covariate in our analysis. While harmonizing imaging data to correct for site effects, variance due to sex was preserved. Model fitting was performed individually for each subject, and sex was used as a categorical covariate in the SVD-PLS analysis comparing model weights with clinical assessments. The distribution of subjects by sex is reported in Supplementary Table S1.
Reporting on race, ethnicity, or other socially relevant groupings	All subjects with sufficient data included in this work were from the same race (Supplementary Table S1).
Population characteristics	Age (mean 59.6 years, s.d. 9.8 years), education (mean 15.5 years, s.d. 2.8 years) and sex (28.2% female) were considered as covariates. Variance due to these covariates was preserved during imaging harmonization, and accounted for in the PLS-SVD analysis. The demographic information is summarized in Supplementary Table S1.
Recruitment	We did not perform recruitment, but used data from Parkinson's Progression Markers Initiative (PPMI). For information about recruitment, please refer to PPMI https://www.ppmi-info.org/study-design/study-cohorts. Participants consisted of patients with a clinical diagnosis of Parkinson's disease and a positive dopamine transporter SPECT imaging scan. Participants were clinically evaluated at baseline and follow-up visits to confirm the diagnosis and exclude other conditions. For more information about recruitment, please refer to https://www.ppmi-info.org/sites/default/files/docs/PA2_PPMI_Clinical%20Protocol_Final_01Feb2021.pdf.
Ethics oversight	PPMI recruited patients at multiple clinical sites. The full list of PPMI sites can be found at https://www.ppmi-info.org/about-ppmi/ppmi-clinical-sites. Following good clinical practices, subjects and/or authorized representatives gave written informed consent at the time of enrollment, and completed questionnaires approved at each participating site by the responsible Institutional Review Boards (IRBs).

Note that full information on the approval of the study protocol must also be provided in the manuscript.

Field-specific reporting

Please select the one below that is the best fit for your research. If you are not sure, read the appropriate sections before making your selection.

☒ Life sciences ☐ Behavioural & social sciences ☐ Ecological, evolutionary & environmental sciences

For a reference copy of the document with all sections, see nature.com/documents/nr-reporting-summary-flat.pdf

Life sciences study design

All studies must disclose on these points even when the disclosure is negative.

Sample size	No sample size calculations were performed. The Parkinson's Progression Markers Initiative (PPMI) dataset was used as it is the largest multi-modal imaging study following Parkinson's disease patients longitudinally. All PPMI subjects with sufficient longitudinal (3 or more imaging visits) and multi-modal (structural, functional and diffusion MRI and DAT-SPECT imaging) data were included. Our modeling approach is longitudinal and multi-modal, requiring three or more imaging visits and all 4 imaging modalities from all included subjects. There were N=71 subjects that fit this criteria and all were included in this study.
Data exclusions	No data was excluded.
Replication	No explicit replication was performed on a separate dataset, due to the lack of similarly multi-modal and longitudinal imaging datasets in PD patients. However, personalized models were fit (individually) for each subject. To identify statistically stable model parameters across

	subjects, 99% confidence intervals were used. Robustness of the association between these model predictors and clinical symptoms was tested using permutation tests and bootstrapping in the PLS-SVD procedure. Additionally, permutation tests were used to evaluate the significance of receptor-informed contributions to each model compared to null models.
Randomization	No randomization was performed as this study does not include experimental groups.
Blinding	Blinding was not performed, as this study does not include experimental groups.

Reporting for specific materials, systems and methods

We require information from authors about some types of materials, experimental systems and methods used in many studies. Here, indicate whether each material, system or method listed is relevant to your study. If you are not sure if a list item applies to your research, read the appropriate section before selecting a response.

Materials & experimental systems

n/a	Involved in the study
☒	☐ Antibodies
☒	☐ Eukaryotic cell lines
☒	☐ Palaeontology and archaeology
☒	☐ Animals and other organisms
☒	☐ Clinical data
☒	☐ Dual use research of concern
☒	☐ Plants

Methods

n/a	Involved in the study
☒	☐ ChIP-seq
☒	☐ Flow cytometry
☐	☒ MRI-based neuroimaging

Magnetic resonance imaging

Experimental design

Design type	T1- and T2-weighted, resting-state fMRI, and diffusion-weighted MRI
Design specifications	All subjects underwent MRI at baseline and follow up scans. For a detailed description of study design and acquisition protocols, please see http://www.ppmi-info.org.
Behavioral performance measures	No behavioral measures were recorded during fMRI.

Acquisition

Imaging type(s)	Structural, functional, diffusion
Field strength	3T
Sequence & imaging parameters	MRI scans were obtained from multiple sites. For a detailed description of acquisition protocols, please see http://www.ppmi-info.org. For resting-state fMRI, acquisition parameters were: 140 time points, repetition time (TR)=2400 ms, echo time (TE)=25 ms, flip angle=80°, number of slices=40, slice thickness=3.3 mm, in plane resolution=3.3 mm and in plane matrix=68×66. T1- and T2-weighted images from 3T scanners were acquired as a 3D sequence with a slice thickness of 1.5 mm or less, under three different views: axial, sagittal and coronal. Diffusion-weighted images were acquired along 64 uniformly distributed directions using a b-value of 1000 s/mm2 and a single b = 0 image. Single shot echo-planar imaging (EPI) sequence was used (116 × 116 matrix, 2 mm isotropic resolution, TR/TE 900/88 ms, and twofold acceleration).
Area of acquisition	Whole brain scanning was performed.

Diffusion MRI ☒ Used ☐ Not used

Parameters	Diffusion-weighted images were acquired along 64 uniformly distributed directions using a b-value of 1000 s/mm2 and a single b = 0 image. Single shot echo-planar imaging (EPI) sequence was used (116 × 116 matrix, 2 mm isotropic resolution, TR/TE 900/88 ms, and twofold acceleration). An anatomical T1-weighted 1 mm3 MPRAGE image was also acquired.

Preprocessing

Preprocessing software	Preprocessing was done using FSL and SPM12.
Normalization	Diffusion-weighted MRI preprocessing steps included: 1) motion and eddy current correction, 2) EPI distortion correction, 2) alignment of the T1-weighted image to the b0 image based on mutual information, 3) calculation of the deformation field between the diffusion and T1-weighted images, 4) calculation of the voxelwise diffusion tensors, 5) alignment to the structural T1 image, and 6) spatial normalization to MNI305 space.

For structural MRI, 1) all images underwent non-uniformity correction using the N3 algorithm, and 2) were segmented into gray matter probabilistic maps using SPM12, which were 3) standardized to MNI305 space using the DARTEL tool.

Resting-state fMRI preprocessing steps included: 1) motion correction, 2) slice timing correction, 3) alignment to the structural T1 image, and 4) spatial normalization to MNI space using the registration parameters obtained for the structural T1 image with the nearest acquisition date, and 5) signal filtering to keep only low frequency fluctuations (0.01–0.08 Hz).

Normalization template: MNI305

Noise and artifact removal: The first 10 temporal volumes were removed to avoid unstable signals. Signals were linearly detrended, and motion parameters were regressed out.

Volume censoring: No volume censoring was performed.

Statistical modeling & inference

Model type and settings: The regional rates of change of six imaging-derived metrics (fALFF, gray matter density, t1/t2 ratio, mean diffusivity, fractional anisotropy, DAT density) were fit as a function of the imaging metrics, 15 neurotransmitter receptor densities and anatomical connectivity using 6 multi-linear regression models (one per imaging metric) per subject. Model parameters were compared with clinical assessments for each subject using PLS-SVD.

Effect(s) tested: We tested whether subject-specific model parameters co-varied with multivariate clinical assessments.

Specify type of analysis: ☐ Whole brain ☒ ROI-based ☐ Both

Anatomical location(s): A brain parcellation based on cyto- and receptor-architecture was used (Supplementary Tabel S4).

Statistic type for inference (See Eklund et al. 2016): No inference was performed.

Correction: No correction was performed.

Models & analysis

n/a	Involved in the study	
☒	☐	Functional and/or effective connectivity
☒	☐	Graph analysis
☐	☒	Multivariate modeling or predictive analysis

Multivariate modeling and predictive analysis: Regional values of fractional amplitude of low-frequency fluctuations (fALFF) from rs-fMRI, gray matter density maps from T1-weighted MRI, t1/t2 ratio from T1- and T2-weighted MRI, fractional anisotropy and mean diffusivity from diffusion-weighted MRI, and DAT density from DAT-SPECT were used to fit 6 multi-linear regression models for each subject, where these imaging-derived metric, their interactions with receptor densities, direct receptor density effects, and anatomical network-weighted propagation are predictors for each model. The output variables are the rates of change of each of the 6 imaging-derived metrics at each region of interest.
